# Evaluation of Forest Recovery over Time and Space Using Permanent Plots Monitored over 30 Years in a Jamaican Montane Rain Forest

**DOI:** 10.1371/journal.pone.0048859

**Published:** 2012-11-14

**Authors:** Shauna-Lee Chai, John R. Healey, Edmund V. J. Tanner

**Affiliations:** 1 Department of Plant Sciences, University of Cambridge, Cambridge, Cambridgeshire, United Kingdom; 2 Ecosystem Management Unit, Alberta Innovates-Technology Futures, Vegreville, Alberta, Canada; 3 School of Environment, Natural Resources and Geography, Bangor University, Bangor, Gwynedd, United Kingdom; 4 Department of Plant Sciences, University of Cambridge, Cambridge, Cambridgeshire, United Kingdom; Michigan State University, United States of America

## Abstract

Conservation of tropical forest biodiversity increasingly depends on its recovery following severe human disturbance. Our ability to measure recovery using current similarity indices suffers from two limitations: different sized individuals are treated as equal, and the indices are proportionate (a community with twice the individuals of every species as compared with the reference community would be assessed as identical). We define an alternative recovery index for trees – the Tanner index, as the mean of the quantitative Bray-Curtis similarity indices of species composition for stem density and for basal area. We used the new index to compare the original (pre-gap) and post-gap composition of five experimental gap plots (each 90–100 m^2^) and four control plots over 24–35 years in the Blue Mountains of Jamaica. After 24–35 years, these small gaps surrounded by undisturbed forest had recovered 68% of the sum of per species stem density and 29% of the sum of per species basal area, a recovery index of 47%. Four endemic species were especially reduced in density and basal area. With the incorporation of basal area and stem density, our index reduces over-estimations of forest recovery obtained using existing similarity indices (by 24%–41%), and thus yields more accurate estimates of forest conservation status. Finally, our study indicates that the two kinds of comparisons: 1) over time between pre-gap and post-gap composition and 2) over space between gap plots and spatial controls (space-for-time substitution) yield broadly similar results, which supports the value of using space-for-time substitutions in studying forest recovery, at least in this tropical montane forest.

## Introduction

A simple and easily calculated recovery index that quantifies the composition of a regenerating community, as compared with either the original composition or the composition of nearby reference sites, would be of great value to studies of succession and restoration. Besides dealing with species presence, such an index would usefully include both the numbers of individuals per species and some measure of the size of the individuals. There are various measures or indices of similarity in the literature, but all suffer from two limitations when used to estimate forest recovery. The first limitation is that different-sized individuals are treated as equal, for example in the Chao index [1],[2] and abundance-based versions of the Jaccard and Sørensen indices. To cope with this problem, authors have used these indices separately on each of three tree size classes when comparing amongst mature and second-growth forests [2],[3]. The second limitation is that existing indices are proportionate, which means that a community with twice the density of individuals of every species as compared with the reference community would be assessed as identical (as in, for example, [4]). We propose an alternative index which combines number of stems and size of stems into a simple, easily calculated Bray-Curtis ‘recovery index’(BC_RI_). – which we call the Tanner index.

Within a timeframe of about 30 years, and depending on certain site-specific characteristics, such as land use history and the availability of seed sources, tropical secondary forest tree communities in Puerto Rico, Costa Rica, Argentina, Colombia and Venezuela have been reported to recover attributes such as stem density and diversity, while other attributes such as species composition recover more slowly [5],[6],[7],[8],[9]. Factors such as land use intensity and duration are known to affect species diversity in forests that re-grow after the abandonment of agriculture [10],[11],[12]. The importance of surrounding old-growth forest in providing seed sources is also a factor which assists forest recovery [3]. This knowledge of recovery has, however, mostly been gained using chronosequence studies, which substitute a one-time study of sites in different stages of succession (space-for-time substitution), for long-term monitoring of forest dynamics in permanent sample plots [7]. The feasibility of the chronosequence approach means that processes such as forest recovery which occur over decades or hundreds of years may be studied rapidly. The drawback to this approach, however, is that site-specific differences are attributed to successional changes, whereas there may be between-site covariates such as land use history, seed availability and environmental conditions that are an important source of variation in addition to forest age [7],[13]. In the Amazon, chronosequence predictions over-estimated secondary forest re-growth and biomass accumulation in young sites (four years old), as land use intensity was a confounding variable – with older sites having undergone less intensive land use, compared with younger sites [14]. In Mexico and Costa Rica, species composition in chronosequence studies was found to be generally more different than in a single site monitored over time in secondary forests of the same age. This occurred up to 16 years in Costa Rica but only three years for the Chiapas, Mexico study site [7].

Capacity for forest recovery from intensive human disturbance is linked to its capacity for recovery after natural disturbance events [15], [16]. Tree communities growing in locations long subject to intensive and frequent natural disturbance can be theorized to have high levels of resilience (‘returning to a reference state’ [17]) due to past selection of which species survive (and become abundant) in the community, as well as natural selection within the populations of these species. Our study was in such a location, the Blue Mountains of Jamaica, which has been long subjected to hurricanes [18]. We investigated recovery in the tree community following experimental disturbances designed to emulate two kinds of disturbance; 1) the most intensive natural patchy impacts of a hurricane on ridge-top sites and 2) forest-clearance by humans. These experiments, with plots 24–35 years old, are amongst the longest duration of any in the world for experimentally disturbed plots in tropical forest, where both the original composition of the plots is known and adjacent control plots are enumerated. Our first objective is to investigate whether our new recovery index produces a better assessment of long-term recovery of forest compared with existing indices. We compare the recovering plots with the original vegetation, and with nearby undisturbed control plots, providing a temporal and a spatial comparison, respectively, to meet our second objective of investigating whether the two give similar measures of recovery.

## Materials and Methods

### Ethics

All necessary permits were obtained for the described field studies. The study took place inside a National Park. We received permission from the National Environment and Planning Agency (NEPA) and Forest Department in Jamaica to conduct the study.

### Study site and permanent sample plots

The study site is montane tropical forest on the western section of the Grand Ridge of the Blue Mountains in Jamaica (18°05′N, 76°39′W) at 1580–1600 m elevation; a detailed description of these forests can be found in Tanner (1977) [19]. The Blue Mountains have been long subjected to natural disturbance in the form of hurricanes and earthquakes [20],[18]. The forest is species poor, for the tropics, because it is on an oceanic island and at high altitude; 33 species accounted for 95% of the basal area and 93% of stems (in 2009); thus our relatively small sample of nine plots each of 90–100 m^2^ area can be used to summarise important processes for the most common species in the forest. The tree species richness originally present in our five experimental gap plots (48) equated to 84% of the 57 species with stems ≥3 cm DBH in the forest area surrounding the study site (comprising the Mor Ridge, Mull Ridge, Wet Slope and Col/Gully forest types described by Grubb & Tanner (1976) [21] and Tanner (1977) [19]).

**Figure 1:**
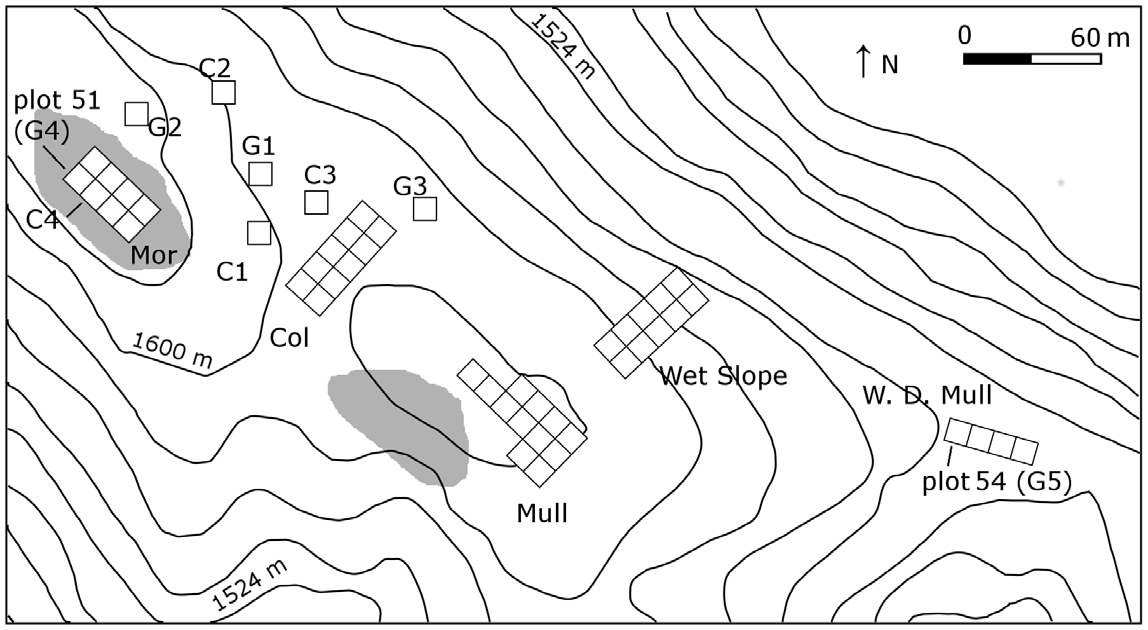
Map showing relative locations of gap (G1–G5) and control plots (C1–C4) in different forest types along the Grand Ridge of the Blue Mountains. W.D. Mull = well developed Mull Ridge forest.

We used four pairs and one single permanent sample plot. Pairs 1–3, each measured 9.5×9.5 m and were on a flat area adjacent to the ridge top which shared characteristics of the Col, Wet Slope and Mull Ridge forest types (the former termed ‘Gap’ forest in Tanner (1977) [19] referring to a “gap” in the profile of the ridge top) (Figure 1). The two plots in each pair were located within 30 m of each other and were purposefully matched in terms of forest structure, basal area and composition. After permanent marking and enumeration of stems, one plot of each pair was randomly selected for experimental gap formation between December 1985 and August 1986, with the other plot left as an undisturbed control [22]. In the gap plots, all stems ≥5 m in height were pulled down to below that height using a winch to simulate the most intense patch effects of strong hurricane winds on ridge-top forest [23]; it is similar to the anthropogenic disturbance of tree harvesting in patches or groups, but without trunk removal and with much less damage to the seedling bank. Most of the larger trees were uprooted and most of the smaller trunks snapped, resulting in mortality of stems (defined as ≥1.3 m in height and ≥3 cm diameter at breast height (dbh)) of 28–30% in the three plots, nine months later. Plot pair 4 and single plot 5 were each 10×10 m, and were in Mor Ridge and well-developed Mull Ridge forest types, respectively. One plot of pair 4 and plot 5 (numbered 51 and 54, respectively, in Tanner 1980 [24]), were converted into gaps in 1975 and 1977, respectively, when all above-ground plant biomass, including seedlings and herbs, was removed for measurement. A matched undisturbed plot adjacent to gap plot 4 was monitored as its control. However, the adjacent control plot for gap plot 5 could not be monitored because its marking was insufficiently permanent and is excluded from this study. In gap plot 4, at least 30 tree individuals survived nine years after gap formation resulting in a mortality rate of approximately 36% [25]. Stem (≥3 cm dbh) mortality rate in gap plot 5 was higher, 64% eight years after gap formation. For both gap plots 4 and 5 the surviving stems had re-sprouted from the cut stumps. This form of disturbance in these two gaps emulates complete small-patch forest clearance by tree cutting (but without subsequent agriculture).

All five gap plots were surrounded by old-growth forest, not subject to any recent human disturbance. The complex land use history of the Blue Mountain forests is discussed in Chai & Tanner (2011) [26]. During the multi-year monitoring period 1975–2010, the forest was affected by Hurricane Gilbert in 1988 [23]. No other storm or hurricane exerted a major impact on the forest since at least 1951.

Forest re-growth in all five gap plots and composition in the adjacent control plots were monitored intermittently for 24 to 35 years, up to 2010. Prior to formation of the five experimental gaps, all stems ≥3 cm dbh (at 1.3 m) were enumerated in the gap and control plots; these enumerations are termed pre-gap and pre-control, respectively. In 2010, we enumerated all stems ≥3 cm dbh (at 1.3 m) present in the five gap and four control plots, termed post-gap and post-control. In addition, 19 surrounding 100 m^2^ plots, ten in Mull Ridge forest and nine in Col forest, were enumerated in 2009 to ascertain spatial variability amongst plots in this forest area.

Plot pairs 1–3 were 90.25 m^2^ in area and 4–5 were 100 m^2^, however this variation does not confound any of the comparisons, because for each gap and control pair the areas were identical. Given that the focus of comparisons is over time in the same plot or between the plots in pairs, and the variation in size amongst pairs is small (<11%), to avoid excessive complication in the calculations, stem number per plot is used as an index of density. Recovery of the tree population was assessed by the Tanner index (BC_RI_) and a range of variables: species composition, species density (species number per plot), stem density and basal area in stems ≥3 cm dbh. Species favoured by gap formation and shade-tolerant species were designated based on independent classifications from previous research in the same general forest area [25],[23], [27] [28], [29],[18]. Nomenclature follows Adams (1972) [30], except where other authorities are given.

### Data analyses

We used the quantitative Bray-Curtis similarity index (BC) to assess the recovery of both stem density and basal area:
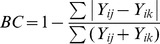
where Y_ij_ and Y_ik_ are the number of stems (or basal area) for each of Y species in sites j and k, respectively. We define our ‘Tanner index’ (BC_RI_) as the mean of the quantitative Bray-Curtis similarity indices for stem density (BC_density_) and basal area (BC_ba_). We chose the quantitative Bray-Curtis similarity index over the Chao-modified Jaccard index [2], Horn index or Horn-Morisita index because the latter are proportionate and therefore cannot distinguish, for example, two plots, one of which has twice as many trees per species as the other. We compared pre-gap and post-gap enumerations (temporal similarity), and post-gap and post-control enumerations (spatial similarity). As gap-plots cannot be expected to ‘recover’ exactly to either their pre-gap condition or that of the controls, we calculated the maximum similarity that could be expected from the temporal and spatial comparisons as (i) the similarity of the pre- and post-control plots and (ii) the similarity of the pre-gap and pre-control plots, respectively. We also calculated plot recovery similarities using the Chao modified-Jaccard [2] and Horn-Morisita (adapted from Horn 1966 [31]) indices as comparators for the Tanner index BC_RI_. All similarity indices were calculated using the R package ‘vegan’ [32],[33]. As a further assessment of the inter-plot spatial variability in species density and basal area in this forest area, we evaluated the similarity amongst 19 plots enumerated in 2009 in two types of forest surrounding our gap experiment (ten in the Mull Ridge forest and nine in the Col forest). Each plot was compared with each of the others in the same forest type separately for stem density, basal area and BC_RI_ (giving a total of 81 pair-wise comparisons using each index); because we repeatedly used the same plot we report mean values for these similarities without error estimates.

We compared changes in species composition, species density (species per area), endemic species density (endemic species per area), stem density (stem number per plot) and basal area (m^2^ ha^−1^) of trees (≥3 cm dbh) between the pre- and post-gap enumerations to investigate the components of forest recovery. The difference in these changes over time between the gap and control plots was assessed using two-tailed paired t-tests. To determine whether differences in plot species richness were due to differences in stem density or actual species richness, we carried out sample-based rarefaction using the EstimateS software [34] to adjust for unequal numbers of stems.

Importance values (IV = (relative basal area+relative density)/2) of each tree species were calculated for the gap and control plots for both the pre- and post-gap enumerations. Indicator species for the forest that re-grew in the gaps were detected using the R package ‘labdsv’ [35], where species are ranked on their value as an indicator on a scale of 0–1 (with 1 being a perfect indicator) based on their concentration within a plot category (gap versus control), and the faithfulness of their occurrence in the plot category. Monte Carlo randomizations were used to test for statistical significance.

## Results

**Figure 2:**
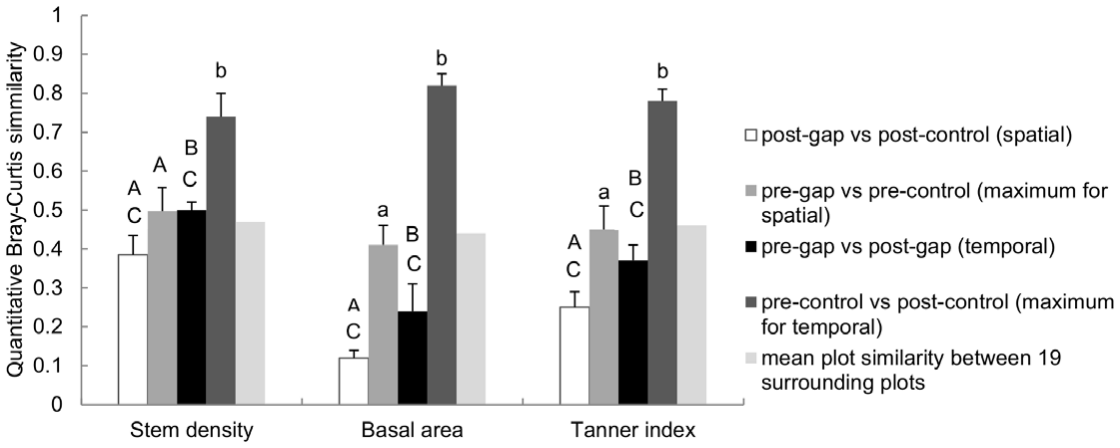
Quantitative Bray-Curtis similarity indices for stem density, basal area and recovery (mean of stem density and basal area) showing the recovery of four gap plots compared with their controls (plot pairs 1–4) and with their pre-gap composition. For comparison, the mean similarity index amongst 19 plots in the surrounding forest in 2009 is also shown. Maximum similarity for the spatial comparison was taken as the mean similarity between pre-gap and pre-control plots and, for the temporal comparison, as the mean similarity between the pre-control and post-control plots. The differences in mean quantitative Bray-Curtis similarity index within each of the three panels were computed using paired t-tests between bars labelled with the same letter; where the letter is shown as lower case it indicates a significant difference from where it is upper case, where both are shown as upper case the difference was not significant.

The recovered forest stem density (sum of per species stem densities) in experimental gaps was similar when compared to control plots (a spatial comparison) or when compared to the original composition (a temporal comparison), but basal area recovery was in both cases significantly less (Figure 2). Using the spatial comparison, for stem density, the gap plots had recovered to a mean similarity of 78% of the estimated maximum spatial similarity attainable (BC_density_ = 0.39/0.50, post-gap versus post-control/pre-gap versus pre-control; Figure 2), but for basal area the plots had only recovered to a similarity of 29% of the maximum (BC_ba_ = 0.12/0.41) which was significantly lower than the maximum. The Tanner index (mean of BC_density_ and BC_ba_) for the spatial (post-gap versus post-control) comparison was therefore 60% of the maximum (BC_RI_ = 0.25/0.45) which was also significantly lower. The temporal comparison, pre-gap versus post-gap, showed a comparable mean recovery to 68% of the maximum for stem density (BC_density_ = 0.50/0.74) and 29% of the maximum for basal area (BC_ba_ = 0.24/0.82), combining to a Tanner index of 47% (BC_RI_ = 0.37/0.78) with all three being significantly lower than their maximum attainable similarity. The spatial comparison of the pre-gap versus pre-control plots was similar for all three indices to the mean plot similarity between the 19 surrounding plots, which shows that our sample was representative. In contrast, the post-gap versus post-control similarity was still lower than for that amongst surrounding plots, especially for basal area (Figure 2).

**Figure 3:**
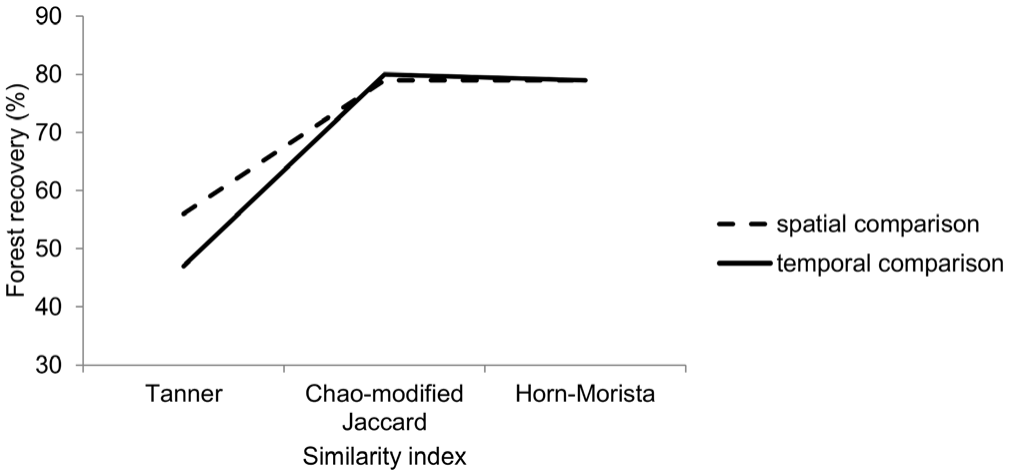
Forest recovery determined by spatial and temporal comparisons measured by the Chao-modified Jaccard index, the Horn-Morista index and the Tanner index.

A comparison of forest recovery in our plots between existing similarity indices of species composition based on stem density and the Tanner index BC_RI_ generally showed higher rates of forest recovery using the existing indices than using our BC_RI_ (Figure 3). The Chao-modified Jaccard index yielded no significant difference between the post-gap plots and the post-control plots (spatial comparison) versus the estimated maximum for this comparison (79%; 0.61 versus maximum 0.77) or between post-gap plots and their pre-gap composition (temporal comparison; 80%; 0.72 versus maximum 0.90). The Horn-Morisita index produced similar results: no significant difference between the similarity of post-gap and post-control plots (spatial comparison) versus the estimated maximum (79%; 0.46 versus maximum 0.58), however it did show that the post-gap and pre-gap similarity (temporal comparison) was significantly lower than the estimated maximum (79%; 0.68 versus maximum 0.86, P = 0.04).

Five species (excluding the non-native invasive *Pittosporum undulatum*) were significant (P<0.05) indicators of gaps. These were: *Brunellia comocladiifolia* (0.8; an obligate gap-demander [25], [28]), *Myrsine coriacea* (0.7; a gap-benefitting species [29]), *Ocotea patens* (Sw.) Nees (0.7; gap-benefitting, but also observed to be shade tolerant [28]), *Critonia paviflora* DC. (0.7; gap-benefitting [27]) and *Miconia quadrangularis* (0.6; near-pioneer [28]). There were some species present in the pre-gap enumeration that experienced notable declines or were completely absent in the post-gap enumeration. Species which experienced the greatest decline in stem density and basal area were: *Haenianthus incrassatus* (which changed from 85 stems ha^−1^and a basal area of 6.1 m^2^ ha^−1^ to being absent), *Podocarpus urbanii* Pilger (from 191 stems ha^−1^ and 5.2 m^2^ ha^−1^ to 42.5 stems ha^−1^ and 0.1 m^2^ ha^−1^), *Lyonia octandra* (from 382 stems ha^−1^ and 4.1 m^2^ ha^−1^ to 127 stems ha^−1^ and 0.2 m^2^ ha^−1^) and *Solanum punctulatum* (from 85 stems ha^−1^ and 3.6 m^2^ ha^−1^ to being absent). These four species are endemic to Jamaica, and all but *Solanum punctulatum* are confined to the Blue and John Crow Mountains ranges in the eastern section of the island [30]. Three of the four species have previously been classified as gap-benefitting, because their *seedling* densities were higher in gaps than under canopy shade, and *Lyonia octandra* as a slow-growing pioneer [27],[29]; however their seedlings which did occur in the gap plots of this study [25],[22] mostly failed to grow into saplings or trees.

There was a heavy invasion by *Pittosporum undulatum*, a gap-benefitting species introduced from Australia [36], in the post-gap plots (with the exception of plot 4 in the distinctive Mor Ridge forest, which, as a whole, is lightly invaded by this species). In the pre-gap enumeration, *Pittosporum undulatum* was absent from the tree and sapling size classes (stems>3 m in height), though it did have seedlings (about 0.5 m^−2^; stems<3 m height; [22]. However, in the 2010 enumeration, 24–35 years after gap formation, it had 118 tree stems (≥3 cm dbh) in total in the 471 m^2^ of the five gap plots (giving a mean density of 0.25 m^−2^) and a total basal area of 16 m^2^ ha^−1^. The species had thus become dominant in the gaps, with an importance value four times higher than any other species (Table 1).

**Table 1:** The ten woody species with the highest importance values (IV) = {(relative density+relative basal area)/2}; stems ≥3 cm dbh in gap and control plots pre- and 24–35 years post-gap formation.

Pre-gap	IV (%)	Post-gap	IV (%)	Pre-control	IV (%)	Post-control	IV (%)
Alc lat*	7.2	Pit und*	38.9	Lyo oct*	13.6	Lyo oct*	14.0
Cle occ*	6.6	Alc lat*	9.5	Cha glo*	9.3	Cha glo*	10.7
Lyo oct*	6.1	Bru com*	6.6	Cle occ*	7.9	Cle occ*	6.8
Hed arb*	5.9	Eug vir+	3.9	Pod urb*	7.2	Cyr rac*	6.5
Cyr rac*	5.5	Hed arb*	3.6	Cyr rac*	5.3	Alc lat*	6.0
Pod urb*	5.4	Cry rac*	3.2	Alc lat*	5.0	Eug vir+	4.6
Hae inc*	5.2	Myr cor*	2.9	Eug vir+	4.9	Hae inc*	4.3
Eug vir+	5.0	Ile mac*	2.8	Clu hav+	4.3	Gua gla+	4.0
Eug mon+	3.9	Mic dod*	2.7	Hae inc*	4.2	Clu hav+	3.7
Sol pun*	3.4	Oco pat*	2.6	Gua gla+	3.9	Pod urb*	3.6

Regeneration type is restricted to a two-class categorization from previous research (based on seedling population distribution and response to disturbance):

* species favoured by gap formation,

+ shade-tolerant species. Alc lat = *Alchornea latifolia*, Bru com = *Brunellia comocladiifolia*, Cha glo = *Chaetocarpus globosus*, Cle occ = *Clethra occidentalis*, Clu hav = *Clusia havetioides*, Cyr rac = *Cyrilla racemiflora*, Eug mon = *Eugenia monticola*, Eug vir = *Eugenia virgultosa*, Gua gla = *Guarea glabra* Vahl., Hae inc = *Haenianthus incrassatus*, Hed arb = *Hedyosmum arborescens*, Ile mac = *Ilex macfadyenii*, Lyo oct = *Lyonia octandra*, Mic dod = *Miconia dodecandra*, Myr cor = *Myrsine coriacea*, Oco pat = *Ocotea patens* (Sw.) Nees, Pit und = *Pittosporum undulatum*, Pod urb = *Podocarpus urbanii* Pilger, Sol pun = *Solanum punctulatum*.

The changes in stem density, species density and endemic species density between the pre- and post- gap enumerations were not significantly different in the five gap plots from the changes in the four control plots (Table 2). However, there was a significant reduction in basal area in the gap plots compared with a slight increase in the control plots. The larger number of stems in the post-gap plots compared with the pre-gap plots did not result in a significantly greater number of species.

**Table 2:** Woody species abundance (mean ± standard error for each plot category) in five gap plots and four control plots pre- and 24–35 years post-gap formation (Pit und = *Pittosporum undulatum*).

Variable	Pre-gap	Post-gap	Pre-control	Post-control	T-test
Stem density (no./90 or 100 m^2^)	55±4	70±2	52±8	60±14	n.s.
Stem density excluding Pit und	55±4	47±8	51±8	57±14	n.s.
Species density (no./90 or 100 m^2^)	18±2	17±2	17±2	18±3	n.s.
Endemic species density (no./90 or 100 m^2^)	9±1	6±1	8±0.4	7±1	n.s.
Basal area (m^2^ ha^−1^)	66.9±5.5	37.2±4.5	58.2±8.7	63.1±10.8	*
Basal area without Pit und (m^2^ ha^−1^)	66.9±5.5	20.7±5	58.2±8.7	62.3±11.4	***

The statistical test is the difference in the *change* from pre- to post- enumerations between the gap and the control plots using a two-tailed paired t-test (n.s. indicates no significance;* P≤0.05; *** P<0.001).

## Discussion

Spatial comparisons are the most common method used to assess recovery from disturbances as few studies have the data available for long-term temporal comparisons. Our long-term study showed broadly similar rates of recovery when judged over space (60% recovery) or when judged over time (47% recovery) using the Tanner Index. Thus, in this forest, a space for time substitution would yield a more optimistic though broadly comparable assessment of recovery.

The observed tree species abundance shows that the forest regenerating in experimental gaps was composed of a mixture of tree species abundant in the previous old-growth forest, and those that had been comparatively rare or even absent. Excluding the recently invasive *Pittosporum undulatum*, of the combined importance values of the top nine species in the post-gap plots, 53% was contributed by the four species that had been present in the top ten pre-gap and 47% was comprised by the five species that were rarer pre-gap. Of the latter five species, two (*Brunellia comocladiifolia* and *Miconia dodecandra*) were absent as seedlings from gap plots 1–3 before gap formation and were extremely rare as trees or seedlings in the surrounding undisturbed old-growth forest at the start of the experiment. They are strongly gap-benefitting and are the species in this flora that are closest to the class of ‘pioneers’ as defined by Swaine and Whitmore (1988) [37]. The other eight of the ten most abundant species post-gap had all been present as seedlings pre-gap or resprouted from the tree stems cut or broken during gap formation. Thus, forest regrowth in gaps in this montane forest is characterised by a mixture of “release” of growth of species already present as seedlings and saplings, and those that germinated only after gap formation, a parallel situation to that in lowland rainforests in South East Asia [38],[39].

The Tanner index of recovery reduces (by 24–41%) the risk of over-optimistic estimations of the extent of forest recovery based on stem densities alone, as employed by a range of existing indices. The addition of basal area to quantitative indices of forest recovery is important because recovery of basal area in tropical secondary forests is highly variable. Some studies report that the basal areas of 20–40 year-old post-agriculture secondary forests in lowland wet areas are comparable to those of old-growth forest (e.g. [40],[6],[41],[9]), but in our study of recovery in small tree-fall/cut gaps in montane tropical forest, we observed a 44% (69% without *Pittosporum undulatum*) decline in total basal area between pre-and 24–35 year post-gap formation enumerations. A number of other studies also report a similarly slow recovery in basal area: in semi-deciduous forest in the lowlands of the Bolivian Amazon, basal area was 44% lower in 22–36 year-old secondary forest compared with old-growth forest [42], a mid-elevation (1100–1360 m) forest in the Dominican Republic had a 27% lower basal area 40 years after agricultural abandonment compared with old-growth forest, which was attributed to the extended period of agricultural land use during which remnant trees were eliminated [16]. Forests in Western Amazonia had a 30% lower basal area 40 years after slash-and-burn agriculture, which was attributed to poor soils in the region; it was estimated to require 190 years for recovery of old-growth forest basal area [5].

Re-growth of forest after disturbance is therefore dependent on numerous site-specific factors [43] such as duration and intensity of land use, soil fertility, availability of seed sources and dispersal agents, remnant trees, subsequent disturbance regimes (e.g. due to periodic hurricanes [44]), invasion of alien species [36] and growth rate of forest vegetation, which is slower in montane than in lowland sites [45]. In our study, the significantly lower basal area after 24–35 years of forest regeneration following intensive patch disturbance, compared with old-growth forest, can be attributed to the nutrient-poor soils and slow growth rate of trees in this montane site [19],[46], and paucity of surviving remnant trees, despite initial re-sprouting from the main trunks of some snapped stems and coppice growth of some cut stems. This further illustrates the value of measuring tree community recovery in forests on the basis of both density and basal area (as used by the Tanner index). This approach will be advantageous in research studies seeking to understand the role of disturbance in determining forest community dynamics (e.g. [38], [47]), and the basis of the resilience of forest ecosystems to anthropogenic impacts (e.g. [3]). It will also have important applications in monitoring work carried out to assess sustainable forest management, e.g. for forest certification [48], for “Reduced Emissions from Deforestation and Forest Degradation” (REDD+) projects [49],[50] or other payment for ecosystem services projects [51].

### Factors affecting forest recovery in our plots

In addition to the long-term site factors discussed above, there were three important factors that specifically influenced forest re-growth in our gap plots: the short duration of the disturbance (trees cut or winched down in a single event with no subsequent agricultural land use); the presence of surrounding old-growth forest; and hurricane disturbance in both gap and control plots. In 1988 Hurricane Gilbert (strength category 4) caused a stem mortality of 7.2% and severe canopy damage in plots in the surrounding forest [23]. This hurricane affected the control plots of the present study more than the gap plots because vegetation in the gaps was comparatively short when the hurricane hit. In Puerto Rico, hurricane disturbance is known to have a homogenizing effect on both structure [52] and species composition [53] in secondary and relatively undisturbed forest. Hurricanes caused older forests in Puerto Rico to experience ‘delayed succession’ due to a decrease in basal area of large trees, while in younger forests basal area increased as tree stem density increased through recruitment [52]. In the Luquillo Mountains of Puerto Rico, hurricane disturbance stimulated the regeneration of light-demanding tree species in both relatively undisturbed and secondary forest, resulting in a species amalgamation effect in both forest types [53]. Despite the potential homogenizing effect of the 1988 hurricane in our plots, species composition and basal area of the gap and undisturbed control plots remained distinct 12 years later, and could be attributed to the forest being of old growth status in contrast to the hurricane-impacted forest in Puerto Rico which was still heavily influenced by the legacy of previous land use.

### Conservation implications

We have shown that, in the forest that re-grew in gaps after intense experimental disturbances in Jamaican tropical montane forest, the greatest declines in stem density and basal area were in four endemic species (*Haenianthus incrassatus*, *Podocarpus urbanii* Pilger, *Lyonia octandra* and *Solanum punctulatum*) and there was a heavy invasion by the alien species *Pittosporum undulatum*; both of these phenomena were also noted as occurring following anthropogenic disturbance in other tropical forests by Wright (2005) [54]. Four of the five gaps in the present study were heavily invaded by *Pittosporum undulatum* which became the dominant species (importance value 39%). Although *Pittosporum undulatum* had also invaded the four control plots during the 24–35 year study period, its abundance in them was much lower (importance value 2.9%). In Puerto Rico (Aide et al., 2000) and the Dominican Republic [16], the high abundance of alien species was an important feature of secondary forests; *Syzygium jambos* was the commonest invasive species, becoming dominant in some instances in both islands. Whilst benefitting from disturbance, both *Pittosporum undulatum* and *Syzygium jambos* are shade tolerant with a high capacity to regenerate beneath an intact canopy. This suggests that, once established following disturbance of sufficient intensity, these species are able to out-compete native species with the potential to establish canopy dominance and then maintain it through self-replacement. In Jamaica it is observed that the population of *Pittosporum undulatum* well-established in post-agricultural secondary forest is providing an effective source of propagules from which the species is spreading into nearby old-growth forests facilitated by anthropogenic and hurricane-caused disturbance [55].

As the studied forest has a long history of exposure to severe natural disturbance, and the monitored re-growth in gaps occurred within unfragmented forest, the results indicate that such tropical montane forest recovers slowly from disturbance, especially when exposed to invasive woody species. Given the high rate of forest clearance in the Blue Mountains (1.42% yr^−1^ between 1992 and 2002; [56], the area is therefore very vulnerable to the impacts of humans, and high priority should be placed on forest protection and invasive species control for this ecosystem, which is a site of global conservation priority.
